# Vastus Medialis Obliquus Muscle Morphology in Primary and Recurrent Lateral Patellar Instability

**DOI:** 10.1155/2014/326586

**Published:** 2014-04-29

**Authors:** Peter Balcarek, Swantje Oberthür, Stephan Frosch, Jan Philipp Schüttrumpf, Klaus Michael Stürmer

**Affiliations:** Department of Trauma Surgery, Plastic and Reconstructive Surgery, University Medical Center, Robert-Koch-Street 40, 37075 Göttingen, Germany

## Abstract

The morphology of the vastus medialis obliquus (VMO) muscle in the anatomical setting of an unstable patella has not been described. Therefore, the purpose of this study was to investigate the morphological parameters of the VMO muscle that delineate its importance in the maintenance of patellofemoral joint stability. Eighty-two consecutive subjects were prospectively enrolled in this study. The groups were composed of thirty patients with an acute primary patellar dislocation, thirty patients with recurrent patellar dislocation, and twenty-two controls. Groups were adjusted according to sex, age, body mass index, and physical activity. Magnetic resonance imaging was used to measure the VMO cross-sectional area, muscle-fiber angulation, and the craniocaudal extent of the muscle in relation to the patella. No significant difference was found with respect to all measured VMO parameters between primary dislocation, recurrent dislocation, and control subjects with a trend noted for only the VMO cross-sectional area and the VMO muscle-fiber angulation. This finding is notable in that atrophy of the VMO has often been suggested to play an important role in the pathophysiology of an unstable patellofemoral joint.

## 1. Introduction

Lateral patellar dislocation (LPD) predominantly affects young and physically active adolescents and young adults. Typically, LPD is characterized by an imbalance between the active, passive, and static stabilizers of the patellofemoral joint [1–3]. Marked individual variability in anatomical risk factors has also been described in this patient cohort [4]. In addition, the function of the quadriceps muscles, particularly that of the vastus medialis obliquus (VMO) muscle, has been suggested to play an important role in the stability of the patellofemoral joint, notably with regard to patellar shift, patellar tilt, and the force required to displace the patella laterally [3, 5, 6].

Whereas atrophy of the VMO, imbalance of the VMO/vastus lateralis (VL) strength, and altered neuromuscular timing of the different parts of the quadriceps muscle have all been described in patellofemoral pain (PFP) syndrome [7–10], the literature lacks comparable data in patients with lateral patellar instability. In particular, the stabilizing effect of the VMO in the typical anatomical setting of an unstable patella (i.e., trochlear dysplasia, patella alta, and increased tibial tuberosity-trochlear groove distance) has not been described. Furthermore, it is unclear whether atrophy of the VMO precedes primary LPD or develops secondarily as a consequence of pain inhibition and physical inactivity following recurrent dislocations. Therefore, it appears imperative to ascertain the value of the VMO in the stability of the patellofemoral joint, not only with regard to the typical anatomical conditions observed in LPD but also in light of current concepts that favor nonoperative treatment in primary patellar dislocation, which thereby aim to restore and strengthen the extensor apparatus of the knee joint to prevent further episodes of LPD [11, 12]. Thus, the purpose of this study was to investigate the morphology of the VMO in a cohort of primary and recurrent patellar dislocators as measured by three parameters: muscle cross-sectional area, muscle-fiber angulation, and the craniocaudal extent of the VMO relative to the patella. It was hypothesized that these morphologic characteristics of the VMO are diminished in patients with recurrent LPD, but not in primary LPD, compared to an asymptomatic control group.

## 2. Material and Methods

### 2.1. Participants

After approval by our institutional review board (IRB ref. number 13/5/09), a pilot study was conducted that included eight patients with acute primary LPD, eight patients with recurrent LPD, and eight control patients (male/female 4/4 in each group). A power analysis (free G*Power Software, Version 3.1.3.) revealed that a minimum of eighty-one subjects would be required for an observed power (1-*β* error probability) of 90%. Consequently, a total of eighty-two consecutive subjects were prospectively enrolled in this study. The groups were composed of thirty patients with acute primary LPD, thirty patients with recurrent patellar dislocation, and twenty-two control patients without any medical history related to the patellofemoral joint. Groups were adjusted according to sex, age, body mass index (BMI), and physical activity according to Baecke's questionnaire [13] (Table 1). A diagnosis of LPD was based on the medical history, a thorough clinical examination, and magnetic resonance imaging (MRI) criteria of LPD as previously published [1]. MRI investigations were performed within 10 days after injury in both primary LPD (median 6 days from injury to MRI) and control subjects (median 7 days from injury to MRI) in an effort to minimize bias related to muscle atrophy as a consequence of physical rest after injury. MRI investigations in the recurrent LPD group were performed within a pain free interval.

For all subjects, exclusion criteria were any preexisting knee disorders (except a prior patellar dislocation in the chronic LPD subgroup), any prior knee surgery, fractures of the distal femur or tibial head, multiligament knee joint injury, and MRI performed later than ten days after injury. Traumatic patellar dislocations that occurred as a result of direct trauma to the medial patella or a fall onto the knee joint with concomitant patellar dislocation were also excluded.

### 2.2. Image Evaluation

**Figure 1:**
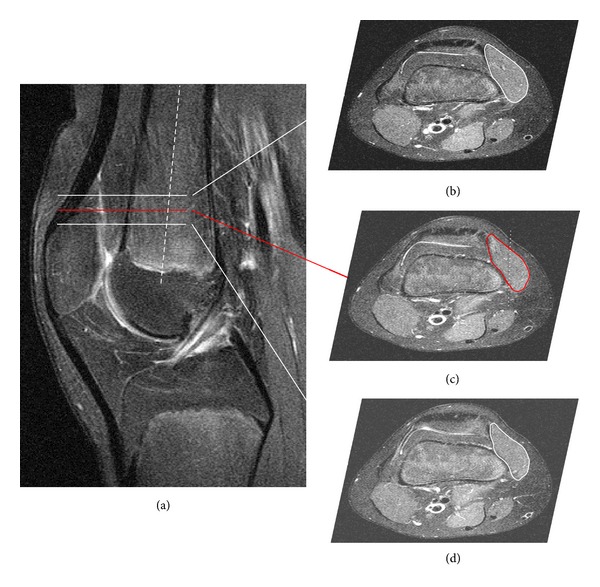
Measurement of VMO cross-sectional area. The longitudinal axis of the patella and the femoral shaft axis (dashed line) were established in the central sagittal plane (a). In this sagittal image, the corresponding transverse slice located at the proximal patellar pole, indicated by the red line (c), and the adjacent slices located above (b) and below (d) this reference slice were identified. These transverse planes were used to measure the VMO cross-sectional area by manually drawing disarticulation contours around the muscle boundaries (solid lines in (b-c)). Additionally, the transverse reference image (c) was used to determine the corresponding sagittal slice located centrally in the VMO muscle (dotted line in (c)).

**Figure 2:**
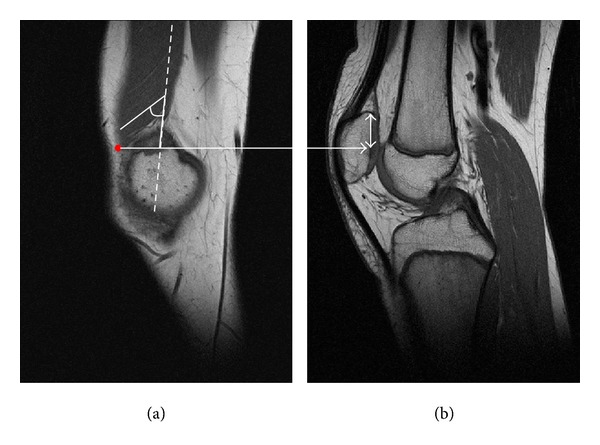
Measurement of VMO muscle-fiber angulation and of the craniocaudal extent of the VMO. This sagittal plane identified by the dotted line in Figure 1(c) was used to measure VMO muscle-fiber angulation. First, the longitudinal axis of the femoral shaft shown in Figure 1(a) was determined in this corresponding plane (dashed line). Muscle-fiber angulation was then assessed in relation to the longitudinal axis of the femoral shaft. To measure the craniocaudal extent of the VMO in relation to the patella, the most caudal end-point of the VMO was determined in a sagittal plane (red dot in Figure 2(a)). This point was then assigned to a corresponding sagittal plane located centrally through the longitudinal axis of the patella (b). The craniocaudal VMO extent was then measured as the distance between this latter point and the proximal patellar pole (double-headed arrow).

Sagittal, coronal, and transverse MR images were obtained in all patients to measure the VMO cross-sectional area, VMO muscle-fiber angulation, and craniocaudal extent of the VMO in relation to the patella. MRI investigations were performed with the knee in full extension and the quadriceps muscle relaxed. Measurements were obtained using the annotation tools of a picture archiving and communications system (PACS) workstation (Centricity, GE Healthcare, St. Gilles, United Kingdom). First, the maximum diameter of the patella and the longitudinal axis of the femoral shaft (dashed line) were established in the central sagittal plane (Figure 1(a)). In this sagittal plane, the corresponding transverse slice located at the proximal patellar pole (red solid line in Figure 1(a)) was identified (Figure 1(c)). Using this transverse image as the reference slice, one trained observer manually measured the VMO cross-sectional area in this slice and in the adjacent slices straight above and below this reference slice (MRI slice thickness 3.5 mm) by drawing disarticulation contours around the muscle boundaries (red solid line and white solid lines in Figures 1(b)–1(d)). All three cross-sectional area measurements were subsumed to one value mimicking the three-dimensional VMO muscle structure. Next, the reference slice in Figure 1(c) was used to determine the corresponding sagittal slice centrally located in the VMO muscle (dotted line in Figure 1(c)). The longitudinal axis of the femoral shaft was assigned to this corresponding plane (dashed line in Figure 2(a)). This sagittal plane, shown in Figure 2(a), was then used to measure muscle-fiber angulation in relation to the longitudinal axis of the femoral shaft. Finally, to ascertain the craniocaudal extent of the VMO in relation to the patella, the most caudal end-point of the VMO was determined in a sagittal plane (red dot in Figure 2(a)). This point was then assigned to the corresponding sagittal plane centrally located through the longitudinal axis of the patella (Figure 2(b)). The craniocaudal VMO extent was then measured as the distance between this point and the proximal patellar pole (double-headed arrow in Figure 2(b)).

Moreover, the main anatomical parameters of LPD (trochlear dysplasia, patellar height, and TT-TG distance) were evaluated as previously published. Trochlear dysplasia was assessed by transverse MRI and classified according to the system described by Dejour et al. [14]. To improve the reliability of the trochlear dysplasia classification, we integrated Dejour's 4-grade classification (Type A–D) into a 2-grade classification system that has recently been recommended: low-grade (type A) and high-grade trochlear dysplasia (types B–D) [15]. Patellar height was evaluated using sagittal T1-weighted images according to the Insall and Salvati index, which is a ratio of patellar tendon length to the longest sagittal patellar dimension [16]. Finally, the TT-TG distance was assessed according to the method of Schoettle [17].

### 2.3. Statistical Analysis

The data are presented as the mean values and standard deviations. Fisher's exact test was used to assess categorical values and an unpaired* t*-test was used to compare the means. A one-way analysis of variance (ANOVA) followed by Dunnett's after test was used to compare the study groups with the control group. To study intra- and interobserver reliability, two measurement series performed on 15 random MRI were drawn either repeatedly by 1 single observer with a 2-week interval or independently by 2 different observers. Reliability was assessed using the correlation (Pearson* r*) between the two measurement series or the mean difference (*t*-test) between these series. All of the analyses were performed using the GraphPad Prism program (version 4; GraphPad Software, San Diego, CA, USA). A *P* value < 0.05 was considered to be significant.

## 3. Results

Demographic data of the study and control groups are presented in Table 1. In comparison to the control group, both primary and recurrent dislocators showed the typical anatomical risk profile of lateral patellar instability with a dysplastic trochlear groove, patella alta, and increased TT-TG distance (Table 2). However, no significant difference was found with respect to all measured VMO parameters between primary dislocation, recurrent dislocation, and control subjects (Table 3). The craniocaudal VMO extent averaged 14 mm in all groups (*P* = 0.957), with a trend noted for only the VMO cross-sectional area and the VMO muscle-fiber angulation between the control and LPD subjects. The control group exhibited a mean increase of 14% and 16% in the VMO cross-sectional area compared to the primary and recurrent LPD groups (*P* = 0.164), respectively, and the VMO muscle-fiber angulation averaged 2° and 4° steeper in the control subjects compared to the values obtained in the primary and recurrent LPD groups (*P* = 0.186), respectively. The intra- and interobserver reliability was highly correlated for all measured parameters, with no significant mean variability observed between all measurement series (Table 4).

## 4. Discussion

The purpose of this study was to investigate, in clinical and anatomical settings of primary and recurrent LPD, the morphological parameters of the VMO muscle that delineate its importance in the maintenance of patellofemoral joint stability. The main findings of this study indicate that VMO morphology does not significantly differ in patients with primary or recurrent patellar dislocation compared to an asymptomatic control group. This finding is notable in that weakness of the VMO has often been suggested to play an important role in the pathophysiology of an unstable patellofemoral joint [18, 19]; further, the restoration of quadriceps strength, in particular the VMO, has been considered an imperative goal to counteract patellofemoral maltracking [5, 20].

Although several anatomical and biomechanical in vitro studies have ascribed the role of active stabilizer of the patellofemoral joint to the VMO muscle [3, 6, 18, 21], clear evidence is lacking regarding its actual stabilizing effect under clinical conditions. Using cadaveric knees, Sakai et al. [6] found an increased lateral patellar shift between 0° to 15° of knee flexion when simulating VMO weakness. Similarly, when the VMO was relaxed, the force required to displace the patella laterally was reduced approximately 30% between 20° and 90° of knee flexion [3]. In the extended knee, however, wherein the patella is least stable, this loss of stability was reduced to only 14%. In addition, trochlear groove geometry and medial retinacular structures, that is, the medial patellofemoral ligament (MPFL), contribute more significantly to the stability of the patella as the knee approaches full extension. Thus, the VMO has not been established as the most important patellar stabilizer in vitro [3]. Indeed, the clinical findings of our study support this previous in vitro assessment in that we did not observe a significant difference between the control and test subjects in all VMO parameters measured. In addition, our study data indicate that proximal soft tissue realignment procedures that aim to strengthen the stabilizing effect of the VMO may often fail to address the main pathology of LPD in patients with anatomical predisposing factors. To some extent, our finding may offer an explanation for why those extra-anatomic techniques yield relatively high rates of redislocation whilst increasing medial patellofemoral pressures [22, 23].

The muscle cross-sectional area is indicative of the force-producing capability of a muscle and can be reliably measured by MRI [24, 25]. In addition, the VMO tension that applies medially and posteriorly may also be influenced by VMO muscle-fiber angulation and the muscle's craniocaudal extent. In previous studies, VMO muscle-fiber angulation has been shown to range between 42° and 52° [6, 18, 26]. These data are in accordance with the 48° ± 8° muscle-fiber orientation observed in our control group. Although not reaching statistical significance, the muscle-fiber angulation in the primary and recurrent LPD groups was, on average, 2° and 4° flatter, respectively. It is unclear whether this finding represents a preexisting characteristic of LPD or a posttraumatic condition. However, the distal parts of the VMO are closely linked to the MPFL. Thus, some authors argue that an injury of the MPFL at its femoral origin is often accompanied by damage to the VMO, which is torn progressively in a proximal direction, thereby losing its correct transverse orientation [27, 28]. It has been suggested, therefore, that MPFL repair should also include the reattachment of the VMO distally to the adductor magnus tendon [29].

This study aims to provide a more detailed analysis of the anthropometric characteristics of the VMO muscle in lateral patellar instability. To the best of our knowledge, this is the first study to assess the morphology of the VMO in primary and recurrent patellar dislocation. The study also correlates this information with the typical clinical and anatomical setting of LPD. The results obtained from this investigation indicate that in the clinical setting of LPD, the VMO muscle plays only a subordinate role in the complex interplay between the different stabilizers of the patellofemoral joint. These findings are in agreement with those of recent studies that point to a shift away from the prior tenets that upheld the restoration of quadriceps strength and function as imperative to successful recovery in PFP syndrome [30–32]. Nonetheless, the results of this investigation should be interpreted within the limitations of the study. First, we measured three morphological parameters of the VMO to be indicative of the muscle's force-producing capability. However, VMO insufficiency may also be traced to a dysfunction of neuromuscular timing or an imbalance between the VMO and the VL. Therefore, we cannot exclude the role of such other factors in patellofemoral instability. Because patients are typically not aware of impending patellar dislocation, it is not feasible to perform electromyography prior to a first episode of LDP. Moreover, a recent study that used a muscle functional MRI evaluation method failed to demonstrate an altered muscle activation pattern in patients with PFP [30]. In the current study, it was not feasible for investigators to be blinded as to which images were obtained from control subjects or patients with LPD, which was further underscored by the presence of multiple imaging findings associated with LPD. Notably, the control subjects were not in optimal musculoskeletal health. MRI investigations performed due to acute injury indicated a meniscal tear in 4 patients, anterior cruciate ligament injury in 14 patients, posterior cruciate ligament injury in 1 patient, and no relevant pattern of injury in 3 patients. None of the subjects in the control group complained of knee-related problems prior to the time of injury, and none reported problems related to the patellofemoral joint. In addition, we were not able to calculate the exact VMO muscle volume. Thus, cross-sectional area measurements were performed on three different heights to mimic the three-dimensional VMO muscle structure though not exactly representing VMO muscle volume. Finally, while the groups were adjusted according to sex, BMI, and physical activity, the mean age was 23.9, 19.4, and 21.3 years (*P* = 0.007) in the control, primary LPD, and recurrent LPD groups, respectively. While these age differences reached statistical significance, we doubt that such differences introduced meaningful bias to the results given satisfactory adjustments for the other three parameters of sex, BMI, and physical activity.

## 5. Conclusion

The findings of this study indicate that VMO morphology does not significantly differ in patients with primary or recurrent patellar dislocation compared to asymptomatic controls. This finding is notable in that atrophy of the VMO has often been suggested to play an important role in the pathophysiology of an unstable patellofemoral joint.

## Figures and Tables

**Table 1:** Demographics of study population and controls.

	Primary LPD *n* = 30	Recurrent LPD *n* = 30	Controls *n* = 22	*P* value
Sex (male/female)	15/15	15/15	11/11	1.0
Age (years)	19.4 ± 4.1	21.3 ± 4.9	23.9 ± 5.5	0.007
Body mass index (BMI)	24.1 ± 3.7	23.3 ± 2.6	25.1 ± 3.6	0.175
Baecke score	8.1 ± 1.2	8.3 ± 1.3	8.5 ± 1.1	0.411

Distribution of sex, age, body mass index, and physical activity according to Baecke et al. [13] in primary and recurrent lateral patellar dislocations and the control group. Descriptive values are mean ± standard deviation. LPD: lateral patellar dislocation.

**Table 2:** Distribution of predisposing factors of lateral patellar instability.

	Primary LPD *n* = 30	Recurrent LPD *n* = 30	Controls *n* = 22	*P* value
Trochlear dysplasia				
None	1	0	16	<0.001
Mild	9	4	5	
Severe	20	26	0	
TT-TG distance (mm)	13.6 ± 3.3	16.1 ± 4.1	9.0 ± 3.7	<0.01
Patellar height	1.27 ± 0.17	1.29 ± 0.17	1.15 ± 0.11	0.0053

Comparison of trochlear dysplasia, TT-TG distance, and patellar height in primary and recurrent patellar dislocations and controls. Data are presented as frequencies and as mean ± standard deviation. LPD: lateral patellar dislocation; TT-TG: tibial tuberosity-trochlear groove.

**Table 3:** Characteristics of VMO muscle morphology.

VMO	Primary LPD *n* = 30	Recurrent LPD *n* = 30	Controls *n* = 22	*P* value
Cross-sectional area (mm^2^)*	1742 ± 625	1714 ± 572	2034 ± 679	0.164
Muscle-fiber angulation (°)	46 ± 7	44 ± 6	48 ± 8	0.186
Craniocaudal extent (mm)	14 ± 5	14 ± 4	14 ± 3	0.957

Comparison of VMO muscle cross-sectional area, muscle-fiber angulation, and the craniocaudal VMO muscle extent in patients with primary and recurrent patellar dislocations and the control group. Descriptive values are mean ± standard deviation. VMO: vastus medialis obliquus; LPD: lateral patellar dislocation; *sum of measured values (three transverse slices).

**Table 4:** Intra- and interobserver reliability of VMO measurement series.

	Pearson *r*	*P* value	Mean of differences	*P* value
Intraobserver reliability				
Cross-sectional area	0.99	<0.0001	5.58	0.36
Muscle-fiber angulation	0.97	<0.0001	0.1	0.9
Craniocaudal extent	0.97	<0.0001	0.17	0.71

Interobserver reliability				
Cross-sectional area	0.99	<0.0001	18.17	0.1
Muscle-fiber angulation	0.84	0.0003	0.56	0.72
Craniocaudal extent	0.92	0.0001	−0.33	0.44

Correlation and mean of differences between 2 measurement series on the same 15 individuals, drawn repeatedly by 1 single observer and 2 different observers.
